# Medical Items

**Published:** 1895-06

**Authors:** 


### *MEDICAL ITEMS.*

FLORIDA loses one of her best physicians in the death of Dr. John P. Wall, of Tampa. He was in attendance upon the State Medical Association at Gainesville, and died suddenly (April 16) while reading his paper on "Public Hygiene." Dr. Wall was about fifty-eight years of age, and highly respected in his profession.

---

THE *American Lancet*, of Detroit, has suspended voluntarily. Its successor seems to be *Medicine*, issued from Chicago by the publisher of the *Lancet*, and edited by Dr. Harold H. Moyer. The first two numbers of *Medicine* have reached us, indicating a very creditable beginning. They contain several excellent articles by well known men, and the mechanical make-up of the journal is attractive and tasteful. The journal seems to have already earned a permanent place in medical journalism.

---

IT now develops that the comments made by several journals (this one included) upon a late decision of the Kansas courts were somewhat premature and without foundation. In the murder case in which hypnotism was said to have been used by the defense the conviction of Gray was reached by other evidence and the element of hypnotic influence over McDonald formed no part of the trial. The presiding judge has emphatically denied the current reports of the case. We are glad to acknowledge the correction.

---

THE chancellor of the University of Mississippi wishes to establish a medical college somewhere in that State for the purpose of "advancing medical education." We desire to assure the honorable chancellor that if he is really anxious to see the cause of medical education advanced he could not advocate a method less likely to accomplish this happy result than the addition of another college to the surplus now existing. *Medical education is not advanced that way.* The sooner that idea can be embraced by the minds of some people the better it will be for medical education and the medical profession.

---

SIMULTANEOUSLY with the late defeat of the Amick Company in its suit against Dr. Reeves, we learn of the reduction of the company's capital stock from $200,000 to $60,000. Dr. Reeves now proposes to publish the entire trial proceedings, with the judge's charge, etc. By the way, since the jury in this case "acquitted Dr. Reeves and virtually admitted that the Amick concern were quacks and their pretended cures fraudulent claims," some of our contemporaries might now explain why they are advertising this quack consumption cure concern. One of them (the *Southern Clinic*, Richmond) is already out with an explanation, rather lame, but the best it could make, doubtless. In the meantime, *he who excuses accuses.*

---

WE are getting somewhat tired of seeing pieces which appeared originally in this JOURNAL copied elsewhere without one particle of credit. In October, 1893, we published some lines entitled "The Medical Raven," by Dr. R. L. Goodbred, of Florida, and we have since seen it quoted half a dozen times and accredited to some other journal. The latest offense, however, is by the *Doctor's Factotum*, a trade sheet in New York. Its last issue contains *verbatim* "The Doctor's Wooing," which we published in January, from the pen of Mr. Orr. It also contains "The Medical Raven," credited to the *Medical and Surgical Reporter*, of Philadelphia. Anything that is worth reproducing is worth acknowledging, to say nothing of the principle involved.

